# Diagnostic accuracy and usefulness of the Genotype MTBDRplus assay in diagnosing multidrug-resistant tuberculosis in Cameroon? a cross-sectional study

**DOI:** 10.1186/s12879-017-2489-3

**Published:** 2017-05-31

**Authors:** Ngu Njei Abanda, Josiane Yvonne Djieugoué, Eunjung Lim, Eric Walter Pefura-Yone, Wilfred Fon Mbacham, Guy Vernet, Veronique Mbeng Penlap, Sara Irene Eyangoh, Diane Wallace Taylor, Rose Gana Fomban Leke

**Affiliations:** 1 Biotechnology Centre, University of Yaounde I, PO Box: 3851, Yaounde, Cameroon; 2 Department of Tropical Medicine, Medical Microbiology and Pharmacology, University of Hawaii, Honolulu, HI 96813 USA; 3 Mycobacteriology Service, Centre Pasteur of Cameroon, PO Box: 1274, Yaounde, Cameroon; 4 Office of Biostatistics and Quantitative Health Sciences, University of Hawaii, Honolulu, HI 96813 USA; 5 Pneumology service, Yaounde Jamot Hospital, P.O Box: 4021, Yaounde, Cameroon; 6 The Biotechnology Centre, University of Yaoundé 1, BP 8094 Yaoundé, Cameroon; 7 Virology Service, Centre Pasteur of Cameroon, P.O. Box 1274, Yaoundé, Cameroon; 8 Laboratory for Tuberculosis Research (LTR), Biotechnology Centre (BTC)-Nkolbison, University of Yaoundé I, Yaoundé, Cameroon; 9 Laboratory of Immunology and Parasitology, the Biotechnology Centre, University of Yaoundé 1, Yaoundé, Cameroon

**Keywords:** Multidrug-resistant tuberculosis (MDR-TB), Genotype MTBDRplus assay, Pulmonary tuberculosis, Cameroon. *InhA* promoter mutation, *KatG* codon 315 mutation, *rpoB* mutations

## Abstract

**Background:**

Drug-resistant tuberculosis, especially multidrug-resistant tuberculosis (MDR-TB), is a major public health problem. Effective management of MDR-TB relies on accurate and rapid diagnosis. In this study, we assessed the diagnostic accuracy of the Genotype MTBDRplus assay in diagnosing MDR-TB in Cameroon, and then discuss on its utility within the diagnostic algorithm for MDR-TB.

**Methods:**

In this cross-sectional study, 225 isolates of *Mycobacterium tuberculosis* cultured from sputum samples collected from new and previously treated pulmonary tuberculosis patients in Cameroon were used to determine the accuracy of the Genotype MTBDRplus assay. We compared the results of the Genotype MTBDRplus assay with those from the automated liquid culture BACTEC MGIT 960 SIRE system for sensitivity, specificity, and degree of agreement. The pattern of mutations associated with resistance to RIF and INH were also analyzed.

**Results:**

The Genotype MTBDRplus assay correctly identified Rifampicin (RIF) resistance in 48/49 isolates (sensitivity, 98% [CI, 89%–100%]), Isoniazid (INH) resistance in 55/60 isolates (sensitivity 92% [CI, 82%–96%]), and MDR-TB in 46/49 (sensitivity, 94% [CI, 83%–98%]). The specificity for the detection of RIF-resistant and MDR-TB cases was 100% (CI, 98%–100%), while that of INH resistance was 99% (CI, 97%–100%). The agreement between the two tests for the detection of MDR-TB was very good (Kappa = 0.96 [CI, 0.92–1.00]). Among the 3 missed MDR-TB cases, the Genotype MTBDRplus assay classified two samples as RIF-monoresistant and one as INH monoresistant.

The most frequent mutations detected by the Genotype MTBDRplus assay was the *rpoB* S531 L MUT3 41/49 (84%) in RIF-resistant isolates, and the *KatG* S315 T1 (MUT1) 35/55 (64%) and inhA C15T (MUT1) 20/55 (36%) mutations in INH-resistant isolates.

**Conclusion:**

The Genotype MTBDRplus assay had good accuracy and could be used for the diagnosis of MDR-TB in Cameroon. For routine MDR-TB diagnosis, this assay could be used for *Mycobacterium tuberculosis* cultures containing contaminants, to complement culture-based drug susceptibility testing or to determine drug resistant mutations.

## Background

The emergence of drug-resistant tuberculosis, especially multidrug-resistant tuberculosis (MDR-TB), threatens TB control efforts in most countries including Cameroon. MDR-TB is caused by strains of *Mycobacterium tuberculosis (Mtb)* with in-vitro resistance to two of the most potent anti-tuberculosis drugs, Rifampicin (RIF) and Isoniazid (INH) [1, 2]. Recent World Health Organization (WHO) estimates suggest that each year about 460/20882 (2.2%) new pulmonary TB (PTB) patients in Cameroon develop MDR-TB, and another 170/1575 (11%) previously treated PTB patients develop MDR-TB. Unfortunately, only a small fraction (~40%) of these patients have access to drug susceptibility testing (DST) for MDR-TB [3]. In response to the need to expand access to diagnostics for patients at risk of MDR-TB, the government of Cameroon signed an agreement with the EXPAND-TB program (Expand Access to New Diagnostics for TB) project in 2010. The EXPAND-TB program is a UNITAID funded program with the overall objective to increase access to diagnostics for patients at risk of MDR-TB in 27 low-income and high TB burden countries, including Cameroon. In Cameroon, this program plans to strengthen existing MDR-TB diagnostic laboratories and establish new laboratories with the capacity to culture and conduct culture-based DST of *Mtb*. Also, the program introduced the Genotype MTBDRplus assay for the rapid diagnosis of MDR-TB [4].

The Genotype MTBDRplus assay (Hain Lifescience GmbH, Nehren, Germany) is a molecular-based test that can rapidly diagnose MDR-TB. This assay detects MDR-TB by identifying mutations in the *rpoB* gene associated with resistance to RIF and in the *KatG* gene and promoter region of the *inhA* gene associated with INH resistance [5, 6]. Two independent systematic reviews concluded that the Genotype MTBDRplus assay was highly accurate in diagnosing MDR-TB when compared to the culture-based proportion method [7, 8]. These two reviews reported pooled sensitivities of 88.7% [7] and 91% [8] and specificities of 99.2% [7] and 99% [8], respectively, for detection of MDR-TB. Although there is evidence of the accuracy of the MTBDRplus assay, the prevalence of MDR-TB varies widely worldwide. This variation in prevalence of MDR-TB might have a significant impact on the predictive value of the MTBDRplus assay [9]. Thus, it is important to evaluate the assay before its use in routine diagnosis.

In this study, we sought to determine the accuracy of the MTBDRplus assay to diagnose MDR-TB cases in Cameroon. Furthermore, the government of Cameroon has recently adopted, an alternative molecular-based test the GeneXpert MTB/RIF assay (Cepheid, Sunnyvale, CA, USA) for the initial screening of TB patients at risk of MDR-TB [10–12]. The GeneXpert MTB/RIF assay simultaneously detects TB and resistance to RIF directly from clinical specimen within 2 h. At present, the government has introduced the GeneXpert MTB/RIF assay in 3 of the 10 regions of the country but plans to roll-out the assay to all parts of the Country [12]. As such, we discuss on the additional use and role of the Genotype MTBDRplus assay in the diagnosis of MDR-TB amidst the availability of the GeneXpert MTB/RIF.

## Methods

### Study design and setting

Participants were enrolled at six health facilities in three towns in Cameroon, between April 2015 and February 2016. The health facilities were the Jamot Hospital in Yaounde (3.848^0^ N, 11.502^0^ E), the Bamenda Regional Hospital in Bamenda (5.963^0^ N, 10.159°E), and 4 facilities in Douala (4.051^0^ N, 9.768^0^ E) including Laquintinie Hospital, the St Albert the Great Hospital, the dispensary Catholic Barcelone and the New Bell District Hospital. These health facilities are the major TB diagnosis and treatment centers in their respective locations.

### Study participants

Participants aged ≥15 years old visiting any of the six health facilities and diagnosed by the laboratory of the health facility to be sputum smear positive for acid-fast bacilli (AFB) or diagnosed by the consulting clinician at the health facility to have clinical symptoms and signs suggestive of PTB were recruited into the study. This recruitment approach, allowed us to enroll patients from whom sputum samples will likely yield positive *Mtb* culture.

Although there are many naïve PTB patients (*n* = 20,882), very few are MDR-TB (2.2%; *n* = 460/20882). To include a high proportion of patients likely to have MDR-TB, the initial focus was to enrol previously treated PTB patients in whom the proportion of MDR-TB is higher (11%; *n* = 170/1575) and then expand enrollment to include naïve PTB patients. Written informed assent and consent was obtained from all study participants, and a structured questionnaire was used to collect demographic and medical information, including prior PTB history, age, gender, and HIV status. An expectorated sputum specimen was collected from each participant and transported to the study laboratory for mycobacterial culture, DST and MTBDRplus assay.

### Specimen processing and microbiology testing

Mycobacterial culture and drug susceptibility testing were performed at the Mycobacteriology unit of Centre Pasteur du Cameroun in Yaounde. Within 48 h of receipt of sputum samples in the laboratory, smears were made, stained using the auramine staining technique and examined by fluorescence microscopy. The smears were read and graded according to WHO guidelines and that of the International Union against Tuberculosis and Lung Diseases (IUALTD) [13, 14]. The sputum samples were then processed for culturing by decontaminating in Sodium hydroxide-Sodium citrate-N-acetyl L-cysteine (NaOH-NaC-NALC) solution and cultured in MGIT tubes using the BACTEC MGIT 960 system (Becton Dickinson, Franklin Lakes, NJ). MGIT tubes that gave a positive fluorescent signal with the BACTEC MGIT 960 equipment were checked for acid-fast-bacilli using Ziehl-Neelsen staining and confirmed for *Mtb* complex (MTBC) using the TB Ag MPT64 test (SD Bioline, Standard Diagnostics, Suwon, Korea). Cultures growing MTBC were assessed for contamination with other bacteria or fungi by growth on blood agar medium for 24 h at 37 °C. If no contaminants (i.e.*,* Bacteria or fungi) were detected on the blood agar, drug susceptibility testing (DST) was performed using the MGIT 960 Streptomycin-Isoniazid-Rifampicin-Ethambutol (SIRE) kit (Becton Dickinson Diagnostic Systems). If contaminants were detected on the blood agar, the MTBC cultures were decontaminated and the culturing process repeated. The critical concentrations of Streptomycin, Rifampicin, Isoniazid and Ethambutol used in the MGIT 960 SIRE kit were respectively 1.0 μg/ml, 1.0 μg/ml, 0.1 μg/ml and 5.0 μg/ml.

Immediately the DST result of a patient’s sample was available, an aliquot of the culture was made and stored at +4 °C. Once the total number of cultures with DST results reached 226, the stored aliquots were screened using the genotype MTBDRplus assay.

### Genotype MTBDRplus assay

Samples were screened using the genotype MTBDRplus assay according to manufacturer’s instructions [5] and interpreted without knowledge of susceptibility results determined by the MGIT SIRE 960 system. Testing consisted of three steps: DNA extraction using the Genolyse kit (Hain Lifescience, Nehren, Germany), multiplex PCR amplification using biotinylated primers and reverse hybridization. The three steps were carried out in three separated rooms.

Each MTBDRplus strip had 27 reaction zones or probes that hybridize DNA (amplicons). Six of the probes were positive controls, while 21 probes were used to detect resistance to RIF and INH. For the detection of RIF resistance, the strip contained 8 probes that hybridize DNA from codons 506 to 533 of the *rpoB* gene and 4 mutation probes (rpoBMUT1 (D516V), rpoBMUT2A (H526Y), rpoBMUT2B (H526D) and rpoBMUT3 (S531 L)). Similarly, for INH resistance, the strip contains probes that hybridized DNA at codon 315 of the *KatG* gene and positions −1 to −22 on the *inhA* promoter region. The mutation probes associated with INH resistance were katGMUT1 (S315 T1), katGMUT2 (S315 T2), inhAMUT1 (C15T), inhAMUT2 (A16G), inhAMUT3A (T8C), and inhAMUT3B (T8A). When the DNA amplicons hybridized to the probes on the MTBDRplus strip following hybridization, a dark band was produced that was easily interpreted as positive.

The MTBDRplus strips were interpreted in a two-stage process. First, the presence of the 6 control bands was confirmed, demonstrating the assay worked and that MTBC was present. Secondly, susceptibilities to RIF and INH were assessed. A sample was considered to be resistant to the drug if at least one of the wild-type bands was absent or a band indicating a common mutation in the drug resistance-related gene was present. Likewise, a sample was considered sensitive to the drug if all the wild-type bands of the gene were present and no common mutation was detected.

### Statistical methods

Participants were classified as new, previously treated PTB patients or patients with unknown PTB history. Participant characteristics were compared between new and previously treated TB patients using two-tailed Fisher’s exact test or Chi-square test for categorical variables and non-parametric Mann-Whitney test for continuous numerical variables. No adjustment for multiple comparisons was made because only a few planned comparisons were made and the data evaluated are actual observations [15].

To determine the accuracy of the MTBDRplus assay to diagnose MDR-TB, MTBDRplus results were compared to the gold standard MGIT 960 SIRE system. We calculated the sensitivity, specificity, positive predictive value (PPV) and negative predictive value (NPV) with 95% confidence intervals (CI) of the MTBDRplus assay for the detection of MDR-TB and resistance to RIF and INH. Sensitivity was defined as the proportion of isolates correctly determined as resistant by the MTBDRplus assay compared with MGIT 960 SIRE system. Specificity was defined as the proportion of isolates correctly determined as susceptible by the MTBDRplus assay compared with MGIT 960 SIRE system. PPV was defined as the proportion of resistant isolates determined by the MGIT 960 SIRE system among isolates determined as resistant by the MTBDRplus assay. NPV was defined as the proportion of susceptible isolates determined by the MGIT 960 SIRE system among isolates determined as susceptible by the MTBDRplus assay. The degree of agreement between MGIT 960 SIRE system and the MTBDRplus assay was also assessed using Cohen’s kappa (*ĸ*) coefficient. *ĸ* values of >0.75 defined as showing very good agreement, *ĸ* values of <0.4 defined as showing poor agreement and *ĸ* values of 0.4–0.75 defined as showing fair to good agreement [16].

All analyses and comparisons were done with GraphPad prism software, version 6 (GraphPad Software, California, USA) and all results having a *p* < 0.05 were considered statistically significant.

## Results

### Demographic characteristic of the study population

**Fig. 1:**
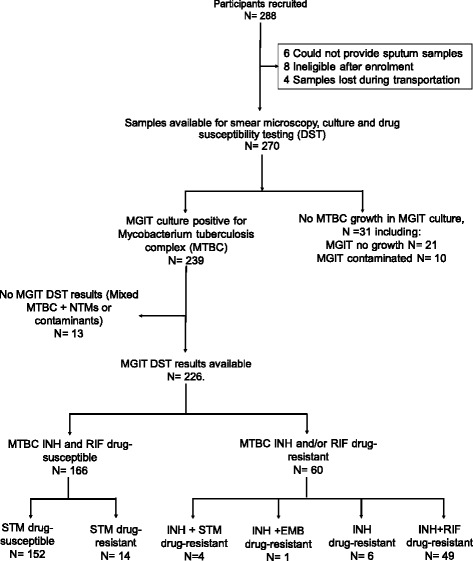
A schematic diagram of sample processing procedure. The Bactec MGIT 960 System was used for cultures and DST. MTBC: *Mycobacterium tuberculosis* Complex; NTMs: Nontuberculous Mycobacterium; INH: Isoniazid, RIF – Rifampicin; STM: Streptomycin; EMB: Ethambutol

Figure 1 shows a schematic diagram of sample processing. Among the 288 participants recruited from 6 health facilities, 270 participants were eligible to participate in the study. Each eligible participant provided a sputum sample that resulted in smear microscopy and MGIT culture data. Of 270 MGIT cultures, 239 samples were positive for MTBC, 21 (7.8%) were negative for growth, and 10 (3.7%) cultures had growth, but the bacteria were not of the MTBC. Of the 239 MGIT-MTBC positive cultures, DST was successfully performed on 226 (95%); Ziehl-Neelsen staining of the 13 isolates without DST results showed that either non-tuberculous mycobacteria or other contaminants like bacteria or fungi had been cultured. Among the 226 isolates with DST results, 152 (67%) were susceptible to both RIF and INH, 49 (22%) were resistant to both RIF and INH, 6 (3%) were INH monoresistant, 5 (2%) were poly-drug-resistant (i.e.*,* to INH plus Streptomycin (*n* = 4) or INH plus Ethambutol (*n* = 1)), and 14(6%) were Streptomycin monoresistant.

Table 1 shows characteristics of the 270 eligible participants. The 169 previously treated TB patients were more likely to be recruited from Yaounde (*p* < 0.0001), more likely to be HIV-negative (*p* = 0.0008), more likely to be sputum smear positive (*p* = 0.0012), and more likely to be drug resistant (*p* < 0.0001) than the 79 new TB patients.

**Table 1:** Demographic characteristic of 270 eligible participants

	All Study participants *N* = 270 (%)	New PTB patients *N* = 79 (%)	Previously treated PTB patients (*N* = 169)	Patients with unknown PTB history (*N* = 22)	^a^ *P*-value
Variables					
Age (y); mean SD	37.7 ± 13.4	36.6 ± 14	38.0 ± 13.5	39.1 ± 10.7	0.271
Gender					0.667
Male	179 (66)	50 (63)	113 (67)	16 (73)	
Female	91 (34)	29 (37)	56 (33)	6 (27)	
Cities					<0.0001
Bamenda	14 (5)	5 (6.3)	9 (5.3)	0 (0)	
Douala	133 (49)	65 (82.3	47 (27.8)	21 (95.5)	
Yaounde	123 (46)	9 (11.4)	113 (66.9	1 (4.5)	
HIV status					0.0008
Positive	63 (23)	13 (16.5)	50 (29.6)	0 (0)	
Negative	140 (52)	40 (50.6)	96 (56.8)	4 (18.2)	
Unknown	67 (25)	26 (32.9)	23 (13.6)	18 (81.8)	
Smear Status					0.0012
Smear negative	44 (16)	5 (6.3)	38 (22.5)	1 (4.5)	
Smear positive	226 (84)	74 (93.7)	131 (77.5)	21 (95.5)	
Grading of positive smears					
*Smear positive (AFB Scanty)*	17 (6)	5 (6.8)	10 (7.6)	2 (9.1)	
*Smear positive (AFB 1+)*	17 (6)	6 (8.1)	8 (6.1)	3 (13.6)	
*Smear positive (AFB 2+)*	46 (7)	22 (29.7)	22 (16.8)	2 (9.1)	
*Smear positive (AFB 3+)*	146 (54)	41 (55.4)	91 (69.5)	14 (63.6)	
Phenotypic DST results					<0.0001
Susceptible	152 (56)	53 (67.1)	82 (48.5)	17 (77.3)	
Mono-drug resistant	20 (7)	7 (8.9	11 (6.5)	2 (9.1)	
Polydrug resistant	5 (2)	2 (2.5)	2 (1.2)	1 (4.5)	
MDR	49 (18)	7 (8.9)	42 (24.9)	0 (0)	
^b^None	44 (16)	10 (12.7)	32 (18.9)	2 (9.1)	

*Abbreviations*: *SD* Standard deviation, *PTB* Pulmonary Tuberculosis, *AFB* Acid-Fast-Bacilli, *DST* Drug susceptibility testing, *MDR* Multidrug-resistant

^a^The *p*-value was obtained from comparison between new and previously treated TB patients individuals

^b^None implies no growth of Mycobacterium tuberculosis complex bacilli observed

### Genotype MTBDRplus test results

The MTBDRplus assay was performed on 225 cultures with MGIT DST results, as one isolate was not recovered following culture of the stored sediment sample. Valid MTBDRplus results were obtained for all 225 cultured isolates on the first attempt. Table 2 provides results comparing the MTBDRplus and MGIT DST assays for the detection RIF-resistant, INH-resistant, and MDR-TB cases.

**Table 2:** Performance of the Genotype MTBDRplus assay in detecting resistance in clinical isolates of Mycobacterium tuberculosis

Gold standard: BACTEC MGIT DST 960 System(# of *Mtb* isolates)							
		RIF		INH		MDR-TB	
Test assay		Resistant	Susceptible	^a^Resistant	Susceptible	Resistant	Susceptible
		(*n* = 49)	(*n* = 176)	(*n* = 60)	(*n* = 165)	(*n* = 49)	(*n* = 176)
MTBDRplus	Resistant	48	0	55	1	46	0
	Susceptible	1	176	5	164	3	176
Sensitivity (95% CI)		98 (89–100)		92 (82–96)		94 (83–98)	
Specificity (95% CI)		100 (98–100)		99 (97–100)		100 (98–100)	
PPV (95% CI)		100 (93–100)		98 (91–100)		100 (92–100)	
NPV (95% CI)		99 (97–100)		97 (93–99)		98 (95–100)	
Cohen’s kappa (95% CI)		0.99 (0.96–1.00)		0.93 (0.88–0.99)		0.96 (0.92–1.00)	

*Abbreviations*: *CI* Confidence interval, *RIF* Rifampicin, *INH* Isoniazid, *MDR-TB* Multidrug resistant Tuberculosis, *PPV* Positive Predictive Value, *NPV* Negative Predictive Value

^a^INH Resistant (*n* = 60) refers to all INH resistant isolates that is; INH and RIF drug-resistant (*n* = 49), INH mono drug-resistant (*n* = 6) and INH poly drug-resistant (*n* = 5)

Overall, the MTBDRplus assay correctly identified RIF resistance in 48/49 (sensitivity, 98% [CI, 89%–100%]), INH resistance in 55/60 (sensitivity 92% [CI, 82%–96%]), and MDR-TB 46/49 (sensitivity, 94% [CI, 83%–98%]). The specificity for the detection of RIF-resistant and MDR-TB cases was 100% (CI, 98%–100%), while that of INH-resistant was 99% (CI, 97% -100%). The PPV and NPV of the MTBDRplus assay were high for RIF resistance, INH resistance and MDR-TB, ranging from 97%–100%. The agreement between both tests for the detection of MDR-TB was very good (Kappa = 0.96 [CI: 0.92–1.00]).

All discordant results between the MGIT DST system and the Genotype MTBDRplus assay are shown in Table 3. The discordant isolates presented with different Genotype MTBDRplus resistant patterns.

**Table 3:** Discordant results between MGIT DST and Genotype MTBDRplus

Patient code	Patient treatment history	MGIT DST				*Genotype MTBDRPLUS assay*			Results
		RIF	INH	STM	EMB	*rpoB*	*KatG*	*inhA*	
HJ128	Previously treated	S	S	S	S	WT	WT	∆WT1, MUT1	INH monoresistant
HJ063	Previously treated	S	R	S	R	WT	WT	WT	Susceptible
HL024	Previously treated	S	R	S	S	WT	WT	WT	Susceptible
HJ088	Previously treated	S	R	S	S	WT	WT	WT	Susceptible
HJ107	Previously treated	R	R	S	R	∆WT8, MUT3	WT	WT	RIF monoresistant
HJ123	Previously treated	R	R	S	S	∆WT8, MUT3	WT	WT	RIF monoresistant
HJ064	Previously treated	R	R	R	R	WT	∆WT1, MUT1	WT	INH monoresistant

*Abbreviations*: *S* Susceptible, *R* Resistant, *WT* Wild Type probe, *MUT* Mutation

∆ Absence of hybridization signal with wild-type probe

### Genotype MTBDR plus mutation patterns

The pattern of mutations associated with INH monoresistant, INH poly-resistant and MDR-TB isolates are shown in Table 4. Overall, the most frequent mutation detected in RIF-resistant isolates was the *rpoB* S531 L MUT3 41/49 (84%). In INH-resistant isolates, the most frequent mutations were *KatG* S315 T1 (MUT1) 35/55 (64%) followed by inhA C15T (MUT1) 20/55 (36%). Majority of the *KatG* S315 T1 mutation was detected among INH polydrug resistant and MDR-TB isolates. Concurrent *KatG* S315 T1 and *inhA* promoter mutations (T8C (MUT3A)) were detected in only one isolate- an MDR-TB isolate.

**Table 4:** Pattern of gene mutations detected by the MTBDRplus assay in 60 drug-resistant Mycobacterium tuberculosis isolates

Gene	Band	Gene region or mutations	^a^INH monoresistant *N* = 6	^a^INH poly-resistant *N* = 5	^a^MDR-TB (*N* = 49)
*rpoB*					
	WT1	506–509	6	5	49
	WT2	510–513	6	5	48
	WT3	513–517	6	5	46
	WT4	516–519	6	5	47
	WT5	518–522	6	5	49
	WT6	521–525	6	5	49
	WT7	526–529	6	5	46
	WT8	530–533	6	5	7
	MUT1	D516V	0	0	0
	MUT2A	H526Y	0	0	0
	MUT2B	H526D	0	0	3
	MUT3	S531L	0	0	41
*KatG*					
	WT	315	5	1	19
	MUT1	S315T1	1	4	30
	MUT2	S315T2	0		0
*inhA*					
	WT1	-15/−16	3	5	32
	WT2	-8	6	5	49
	MUT1	C15T	3	0	17
	MUT2	A16G	0	0	0
	MUT3A	T8C	0	0	1
	MUT3B	T8A	0	0	0

^a^By conventional drug susceptibility testing using the Bactec MGIT 960 system

## Discussion

This study reports the accuracy of the Genotype MTBDRplus assay for diagnosing MDR-TB in new and previously treated PTB patients in Cameroon. Our analysis showed that based on 49 MDR-TB cases, the MTBDRplus assay had a sensitivity and specificity of 94% and 100%, respectively. Such high sensitivity and specificity values make the Genotype MTBDRplus very suitable for use to diagnose MTB-DR in Cameroon. The specificity and sensitivity values reported in our study are similar to those reported in the two systematic reviews of the MTBDRplus [7, 8]. Our participant recruitment strategy led to both a high number of positive *Mtb* cultures and MDR-TB patients (22%; 49/225). This proportion of MDR-TB patients is higher than the population estimate of 2.2% (460/20882) among newly diagnosed TB patients and 11% (170/1575) among previously treated TB patients. However, a sensitivity analysis indicates that the high proportion of MDR-TB patients in our study does not affect the positive predictive value of the Genotype MTBDRplus in diagnosing MDR-TB due to the high specificity value (100%) obtained. Based on our data of 94% sensitivity and an estimated 630 new and previously treated cases of MDR-TB annually in Cameroon, the MTBDRplus assay would fail annually to diagnose 32 cases. These misdiagnosed cases would compromise the goal to identify every patient with MDR-TB in Cameroon. However, among the 3 MDR-TB cases not diagnosed as resistant to RIF and INH by the MTBDRplus assay, 2 were diagnosed as resistant to RIF and 1 as resistant to INH. As such, even if the MTBDRplus assay were to misdiagnose a few MDR-TB cases, the presence of resistance to either INH or RIF among these cases would be detected. Overall, our data suggests that the Genotype MTBDRplus assay can be used to diagnose MDR-TB in Cameroon.

The majority 44/48 (92%) of RIF-resistant isolates detected by the MTBDRplus assay had mutations at codon 531 41/48 (85%) and 526 (6%). These mutations are known to be the most prevalent RIF-resistance associated mutations [17–19], but their frequencies vary worldwide. The frequency of codon 531 mutation in our study was 85% which is higher than generally reported [20]. A similar high frequency of codon 531 mutation among RIF-resistant isolates in Cameroon was also reported [18]. As such, the codon 531 mutation may actually be the most prevalent RIF-resistant associated mutation among RIF-resistant isolates in Cameroon. The relevance of a predominant codon 531 mutations is unclear but could reflect on-going transmission of isolates carrying this mutation. RIF-resistant associated mutation at positions 516 was not detected in this study, but have been reported in other studies in Cameroon [18, 21].

In this study, four RIF-resistant isolates failed to hybridize with one or two of the wild type (WT) probes and did not hybridize with any of the probes representing known mutations. These results suggest that there is either a technical problem or a new previously unreported mutation. The WT probes with no hybridization were mostly WT2, WT3, WT4 and WT8. First, Seifert and colleagues (2016) suggest that this type of result is likely due to the failure of the mutant to hybridize with the mutation probe and is not due to the presence of a rare or new mutation. They concluded that in such situations, improved optimization of the MTBDRplus will demonstrate hybridization to the mutation probes [22]. However, the absence of hybridization could also indicate that there is a mutation at positions 511 (WT2), 516 (WT3), 526 (WT4), and/or 533 (WT8). Mutations at these positions have been reported, but not all mutations have been associated with RIF-resistance [23–26]. In the current study, the four isolates that failed to hybridize to one or two WT probes were RIF-resistant in the MGIT DST, making it likely that unknown mutations associated with RIF resistance could be present. Unfortunately, DNA sequencing could not be done in our study to confirm or identify the mutations; however, since they were resistant by MGIT DST, further studies are warranted.

Besides identifying resistance to RIF or INH, the MTBDRplus assay provides information that is necessary for patient treatment and understanding the evolution of drug resistance. Mycobacterial isolates with the *KatG* codon 315 mutation have reduced ability to activate INH, but the isolate still has catalase and peroxidase activities. As such, isolates with this mutation can persist and be transmitted without any negative selection [27]. Administration of a high-dose of INH (900 mg) per day to patients harboring isolates with the *inhA* promoter mutations, might lead to better treatment outcome [2, 28–30]. Isolates with *inhA* promoter are also resistant to the anti-TB drug Ethionamide (ETH). As such, use of ETH will not be beneficial to the patient [31]. A recent study suggests that routine evaluation of the frequency of the *inhA* promoter mutation can help predict progression to more severe forms of drug resistance. The authors of the study observed an increase in the frequency of *inhA* promoter mutations as isolates progressed to more severe forms of drug resistance, from MDR-TB to pre-extremely drug resistant TB (pre-XDR-TB) and XDR-TB [30, 32].

At present, the WHO recommends that the MTBDRplus assay be used directly on specimen without culturing if the specimen is smear positive and on Mtb isolates obtained after culture [33]. However, among smear-positive specimens, a recent study showed that the MTBDRplus assay will perform best if specimens were graded ≥AFB2+ [34]. In our study, 71% (192/270) of samples were ≥AFB2+. Gauthier and colleagues (2014) have proposed a diagnostic algorithm where the MTBDRplus assay should be used directly on ≥AFB2+ specimens [35], which is appealing because it helps accelerate diagnosis of drug resistance. In reality, a significant proportion of patients will be <AFB2+ and will not be eligible for direct testing according to the algorithm proposed by Gauthier and colleagues (2014). In our study, we had 29% (78/270) of such patients. As such, use of the MTBDRplus assay for direct testing might not be adequate. Furthermore, most laboratories are now equipped with the GeneXpert MTB/RIF assay (Cepheid, Sunnyvale, CA, USA) [11], that performs better than the MTBDRplus for direct testing of clinical specimens [36]. However, the MTBDRplus assay offers great benefit when used on cultured isolates. First, results are available within 48 h in contrast to the 7 days to weeks with culture-based DST. Secondly, the MTBDRplus assay is not affected by the presence of contamination with bacteria, fungi or non-tuberculosis mycobacteria as are most cultured-based DST systems [37, 38]. In our study, 29% (70/239) of the cultures were contaminated and had to be decontaminated and re-cultured before DST could be done. Among the 70 repeats, we were successful in obtaining pure cultures in only 57 cultures; whereas the other 13 remained contaminated and culture-based DST could not be performed. The presence of contaminants increases laboratory processing and reporting times that could be avoided if the MTBDRplus assay is used.

## Conclusions

This study showed that the Genotype MTBDRplus assay has good accuracy for detecting resistance to RIF, INH and MDR-TB, showing it would be useful for the diagnosis of MDR-TB in Cameroon. At present, there are 3 functional TB reference laboratories in Cameroon with culture and molecular-based capacity to diagnose drug-resistant TB. Each laboratory serves health facilities in 3 to 4 regions of the country. The need to transport samples from health facilities to these reference laboratories increases the turnaround time for obtaining results and the risk of having contaminated cultures. The MTBDRplus could be included in the diagnostic algorithm of MDR-TB and be used post-culture. Primarily, the MTBDRplus assay could be used to perform DST of *Mtb* positive-cultures especially for cultures containing contaminants for which culture-based DST will be delayed. Additionally, the MTBDRplus assay could be used as a complementary test to confirm RIF and INH DST results obtained using the culture-based method. Lastly, the MTBDRplus assay can be used for epidemiological surveys to rapidly assess the type RIF and INH mutations present.

## Abbreviations

AFB: Acid-Fast Bacilli
BSL: Biosafety Level
CI: Confidence Interval
DST: Drug Susceptibility Testing
ETH: Ethionamide
EXPANDx-TB: Expand Access to New Diagnostics for TB
INH: Isoniazid
IUATLJ: International Union Against Tuberculosis and Lung Disease
MDR-TB: Multidrug-resistant tuberculosis
MGIT: Mycobacterial Growth Indicator Tube
Mtb: Mycobacterium tuberculosis
MTBC: Mycobacterium tuberculosis complex
MUT: Mutation
NALC-NaOH: N-acetyl-L-cysteine-sodium hydroxide
NIH: National Institute of Health, USA
PTB: Pulmonary Tuberculosis
RIF: Rifampicin
SIRE: Streptomycin, Isoniazid, Rifampicin, Ethambutol
TB: Tuberculosis
WHO: World Health Organization
WT: Wild type.
